# Percutaneous Management of Systemic Fungal Infection Presenting As Bilateral Renal Fungal Ball

**DOI:** 10.1089/cren.2016.0085

**Published:** 2016-09-01

**Authors:** Abhishek Shukla, Nitin Shrivastava, Chirom Amit Singh, Brusabhanu Nayak

**Affiliations:** ^1^ Department of Urology, All India Institute of Medical Sciences, New Delhi, India.; ^2^ Department of ENT, All India Institute of Medical Sciences, New Delhi, India.

**Keywords:** mucormycosis, renal fungal infection, amphotericin B, disseminated mucormycosis

## Abstract

***Background:*** Zygomycoses are uncommon, frequently fatal diseases caused by fungi of the class Zygomycetes. The majority of human cases are caused by Mucorales (genus—rhizopus, mucor, and absidia) fungi. Renal involvement is uncommon and urine microscopy, pottasium hydroxide mount, and fungal cultures are frequently negative.

***Case Presentation:*** A twenty-one-year-old young unmarried lady presented to our emergency department with bilateral flank pain, fever, nausea, and decreased urine output of one-month duration. She was found to have azotemia with sepsis with bilateral hydronephrosis with a left renal pelvic obstructing stone. Even after nephrostomy drainage and broad spectrum antibiotics, her condition worsened. She developed disseminated fungal infection, and timely systemic antifungal followed by bilateral nephroscopic clearance saved the patient.

***Conclusion:*** Although renal fungal infections are uncommon, a high index of suspicion and early antifungal and surgical intervention can give favorable outcomes.

## Introduction and Background

Zygomycoses are uncommon, frequently fatal diseases caused by fungi of the class Zygomycetes. The majority of human cases are caused by Mucorales (genus—rhizopus, mucor, and absidia) fungi. Renal involvement is uncommon, and urine microscopy, potassium hydroxide (KOH) mount, and fungal cultures are frequently negative. Renal mucormycosis usually occurs after disseminated infection. Isolated renal mucormycosis in the absence of infection elsewhere in the body is rare. We present a case where only systemic antifungal with surgical intervention could save the patient after rapid and progressive deterioration.

## Case Presentation

**FIG. 1.**
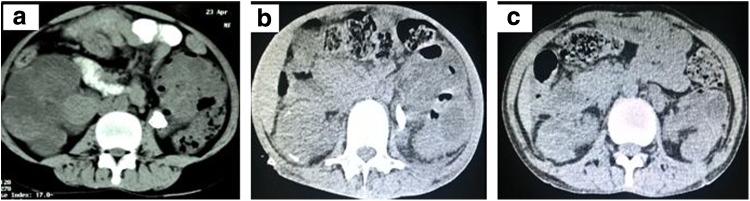
**(a)** Noncontrast computed tomography (NCCT) scan of the abdomen at presentation. **(b)** NCCT scan of the abdomen after amphotericin B therapy. **(c)** NCCT scan of the abdomen after nephroscopic clearance.

In November 2015, a 21-year-old young lady presented to our emergency department with bilateral flank pain, fever, nausea, and decreased urine output for the past 1 month. There was no relevant history. She was febrile (102°F) and tachypneic, with pulse of 110/minute and blood pressure 90/60 mm Hg. Bilateral flank tenderness was noted. Blood investigations revealed anemia (Hb 7.5 g/dL), total leucocytic count of 19,000/dL, blood urea 234 mg/dL, and serum creatinine 19 mg/dL with metabolic acidosis on blood gas analysis. After initial resuscitation, she underwent ultrasound scan of whole abdomen followed by noncontrast computed tomography (NCCT) scan of abdomen that revealed bilateral gross hydronephrosis with left emphysematous pyelonephritis with left renal pelvic stone and right infected hydronephrosis with hyperdense material in the pelvicaliceal system (Fig. 1a).

**FIG. 2.**
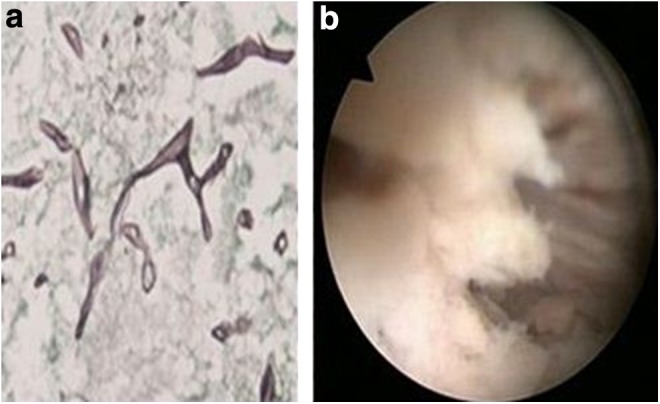
**(a)** Histopathology of the nasal sinus mucosa (silver methenamine stain) showing broad aseptate hyphae. **(b)** Intraoperative nephroscopic view of necrotic material within the pelvicaliceal system.

She was started on broad spectrum parenteral antibiotics, and received hemodialysis with blood transfusion followed by bilateral percutaneous nephrostomy drainage. After showing some initial clinical improvement, her condition continued to deteriorate. Both nephrostomies drained flakes with necrotic debris, for which repeated cultures, microscopy, and KOH mounts were negative. She was extensively evaluated for immunocompromised state, but no underlying cause could be found. She was planned for bilateral nephroscopic removal of stone and necrotic debris, but developed fever and signs of sepsis with metabolic acidosis, and creatinine increased. Despite antibiotics and bilateral wide bore nephrostomy drainage, her condition deteriorated progressively. We had two options, nephroscopic removal of debris and stone or bilateral nephrectomy in an unstable patient or to start systemic antifungal drugs empirically without any microscopic evidence at a creatinine of 3.5 mg/dL and oliguria, on the basis of NCCT findings of hyperdense material in the right pelvicaliceal system. She developed concomitant right maxillary swelling for which nasal endoscopy was done, which revealed necrotic nasal sinus mucosa with necrotic bone. Microscopy of the same was suggestive of mucor (Fig. 2a). Liposomal amphotericin B was then initiated. But by now she was already on mechanical ventilator. She showed dramatic response to amphotericin B and was extubated on day 2 of amphotericin. Nasal debridement and a total cumulative dose of 3.5 g of amphotericin B for 25 days resulted in stabilization of the patient's condition. Her general condition and urine output improved. On repeat NCCT scan of abdomen, both kidneys showed improvement and reduction in parenchymal gas (Fig. 1b). Bilateral sequential nephroscopic removal of stone and necrotic material was done; intraoperative picture is shown in Figure 2b. Postoperative NCCT scan of the abdomen is shown in Figure 1c. She was discharged on bilateral Double-J stents, no external drainage tubes, serum creatinine stable at 2.0 mg/dL, and a urine output of 2.5 L/day. After a 2 months of follow-up, her condition is stable with both the stents removed. Follow-up imaging shows no evidence of residual disease.

## Discussion

Zygomycoses are uncommon, frequently fatal diseases caused by fungi of the class Zygomycetes (consisting of the orders Mucorales and Entomophthorales).

The majority of human cases are caused by Mucorales fungi; therefore, the terms mucormycosis and zygomycosis are used interchangeably. It can be classified as rhinocerebral, pulmonary, cutaneous, gastrointestinal, disseminated, and uncommon rare forms, such as endocarditis, osteomyelitis, peritonitis, and renal infection.^1^ The most common reported sites of invasive mucormycosis have been the sinuses (39%), lungs (24%), and skin (19%). Dissemination develops in 23% of cases.^1^ Renal mucormycosis occurs as a part of disseminated infection in 22% of cases. Isolated renal mucormycosis in the absence of infection elsewhere in the body is rare. There are cases reported in the literature of isolated bilateral renal mucormycosis^2^ and primary renal mucormycosis.^3^ Predisposing conditions include malignant hematologic disease, prolonged and severe neutropenia, poorly controlled diabetes mellitus with or without diabetic ketoacidosis, iron overload, major trauma, prolonged use of corticosteroids, illicit intravenous drug use, neonatal prematurity and malnourishment, and acute and chronic renal failure.^4^ Nosocomial mucormycosis has been seen with exposure to heavy air fungal loads of construction, contaminated air filters, contaminated wound dressings, transdermal nitrate patches, intravenous catheters, and even tongue depressors.^4^ Renal mucormycosis has also been reported in immunocompetent patients in the literature. Diagnosis requires histologic examination of the infected tissue and demonstration of characteristic broad aseptate hyphae branching irregularly at right angles. Blood and urine cultures are often negative. The hyphae are fragile and it is difficult to demonstrate it in the load of necrotic material on a KOH mount. Diagnosis of mucormycosis almost always requires histopathologic evidence of fungal invasion of the tissues. The treatment modalities described are intravenous amphotericin B—cumulative dose of 3 g, surgery alone (debridement of infected tissues, nephrectomy), surgery + antifungal therapy, and various others such as hyperbaric oxygen and granulocyte colony-stimulating factors.^4^ The outcome is affected by the control of the underlying condition, such as diabetes, if any. Mucormycosis has been associated with high mortality rates. The overall mortality rate was 44% in diabetic patients, 35% in patients with no underlying conditions, and 66% in patients with malignancies. The mortality rate varied with the site of infection: 96% of patients with disseminated infections, 85% with gastrointestinal infections, and 76% with pulmonary infections died.^4^

## Conclusion

Disseminated mucormycosis is rare, but high index of suspicion for fungal septicemia and a combined multimodality approach with surgical debridement and systemic antifungal can give favorable outcomes.

## Abbreviations Used

KOH: pottasium hydroxide
NCCT: noncontrast computed tomography
